# Phosphorus supplement alters postprandial lipemia of healthy male subjects: a pilot cross-over trial

**DOI:** 10.1186/1476-511X-13-109

**Published:** 2014-07-08

**Authors:** Jana Hazim, Sani Hlais, Hala Ghattas, Dareen Shatila, Maya Bassil, Omar Obeid

**Affiliations:** 1 Department of Nutrition and Food Science, Faculty of Agricultural and Food Sciences, American University of Beirut, Beirut, P.O. Box 11-0236, Lebanon; 2 Department of Family Medicine, Faculty of Medicine, American University of Beirut Medical Center, Beirut, Lebanon; 3 Department of Natural Sciences, Faculty of Arts and Sciences, Lebanese American University, Beirut, Lebanon

**Keywords:** Phosphorus, Lipids, Lipoproteins, Postprandial lipemia, ApoB48, ApoB100

## Abstract

**Background:**

Epidemiological studies have found a U-shaped relationship between serum phosphorus and cardiovascular disease (CVD). The mechanism(s) behind such a relationship are poorly understood. Phosphorus (P) is reported to improve insulin sensitivity, which is involved in lipid metabolism, and thus we were interested in determining the impact of phosphorus ingestion on postprandial lipemia, a recognized CVD risk factor.

**Findings:**

A within–subject study design was conducted, whereby 8 healthy male subjects received a high fat meal (330Kcal; 69% energy from fat; 35 mg of phosphorus) with placebo or phosphorus (500 mg) in a random order. Postprandial blood samples (~10 ml) were collected every hour for 6 hours after meal ingestion. Changes in different parameters were analyzed using a 2-factor repeated-measure ANOVA. In the phosphorus (P) supplemented group, postprandial serum P increased (p = 0.00), while changes in insulin, non-esterified fatty acids (NEFA) and triglyceride (TG) were not significantly different than that of placebo. Concurrently, phosphorus supplementation increased postprandial concentrations of apolipoprotein B48 (ApoB48) (p < 0.05) and decreased that of apolipoprotein B100 (ApoB100) (p < 0.05).

**Conclusions:**

Phosphorus supplementation (500 mg) of the meal seems to alter the different components of postprandial lipemia. These findings highlight the potential role of phosphorus in CVD.

## Introduction

Postprandial lipemia (PPL) is characterized by the accumulation of triglyceride-rich lipoproteins (TRLs) and their partially hydrolyzed products that results from the absorption of digested dietary lipids in the form of chylomicrons (CM) that contain ApoB48 and/or increased hepatic production of very low density lipoproteins (VLDL) that contain ApoB100 [1]. Elevated serum levels of TRLs or their components (TG, ApoB48 and ApoB100) are reported to be associated with atherosclerosis and cardiovascular disease (CVD) [2] and to be predictors of their future occurrence [3]. In addition, high TG predisposes to other atherogenic risk factors such as low high density lipoproteins (HDL), high small dense low density lipoprotein (LDL) and insulin resistance [4,5]. Hence, PPL reduction is of vital clinical importance and the elucidation of factors able to regulate it can have significant clinical implications for the prevention of atherosclerosis and CVD, particularly as humans are in the postprandial state for a large part of the day. PPL is known to be affected by several factors including diet, lifestyle, genetics, obesity, and insulin resistance [6]. Dietary factors include the amount and type of fat consumed as well as other components of a meal including carbohydrate, protein, alcohol and fiber content [6]. While data on the effect of micronutrients on PPL are limited, magnesium has been reported to improve postprandial lipemia [7].

Both low and high levels of serum phosphorus (P) have been associated with increased risk of CVD [8]. Increased serum P levels have been implicated in CVD morbidity and mortality among subjects with normal and abnormal kidney function [8,9]. The biological mechanism(s) of such an effect are poorly understood, though high P levels were reported to induce vascular calcification and endothelial dysfunction [10,11]. In contrast, low serum P is known to be associated with insulin resistance and impaired glucose tolerance [8], while addition of P to an oral glucose load improves insulin sensitivity [12]. In line with that, serum P status was reported to be inversely related to metabolic syndrome a known risk factor for CVD [13].

It was hypotheses that P addition to a meal would stimulate lipid clearance from circulation due to its capacity to increase insulin sensitivity. Therefore, the nature of the relation between serum phosphate and CVD risk factors is far from clear, which triggered our interest to investigate the effect of P ingestion on insulin and PPL, particularly NEFA, TG, ApoB48 and ApoB100.

## Methods

A total of 8 healthy male subjects participated in the study, since lipid profile is known to be affect by the sex steroids [14]. They had no history of any chronic diseases, including renal, endocrine, hepatic, thyroid, or cardiac disorder. They were not taking any medication that affects body weight, glucose or lipid metabolism. The study protocol was approved by the Institutional Review Board (IRB) at the American University of Beirut. Written informed consent was obtained from all the subjects. The baseline characteristics of the participants are shown in Table 1.

**Table 1:** Main characteristics of the subjects participating in the study

	**Mean ± SEM**
Age (years)	19.25 ± 0.41
BMI (kg/m^2^)	22.72 ± 0.94
Waist circumference (cm)	72.63 ± 3.22
Total phosphate (mg/dl)	3.950 ± 0.18
Glucose (mg/dl)	79.75 ± 1.73
Insulin (μIU/ml)	9.290 ± 1.38
Triglycerides (mg/dl)	100.9 ± 12.9
NEFA (mmol/dl)	0.630 ± 0.12
Apo-B48 (ng/ml)	630.1 ± 21.1
Apo-B100 (μg/ml)	708.9 ± 47.5
Total cholesterol (mg/dl)	142.0 ± 6.38
LDL (mg/dl)	83.70 ± 4.76
HDL (mg/dl)	38.13 ± 2.81

Values are the mean ± SEM. BMI, Body Mass Index; NEFA, Non Esterified Fatty Acids; Apo-B48, apolipoprotein B48; apo-B100, apolipoprotein B100; LDL, Low-Density Lipoprotein; HDL, High-Density Lipoprotein.

### Experimental protocol

The study employed a within–subject design where each participant served as his own control. Experimental sessions were scheduled in random order and were separated by a washout period of a minimum of 1 week. Following 12–14 hours overnight fast, a Teflon catheter was inserted into the antecubital vein in the morning and subjects were asked to remain in a semi recumbent position during the experimental session. They were allowed bathroom access and provided with drinking water, when needed. A baseline blood sample (10 ml) was collected before receiving the treatments (meal with the allocated supplement) which was ingested within 10 minutes. The meal was composed of bread roll (25 g), unsalted spreadable butter (30 g), and jam (14 g) with tablets containing either 500 mg phosphorus or a placebo (Nutricap Labs, Farmingdale, NY, USA). The meal provided 330 Kcal; (69% from fat, 28% from carbohydrate and 3% from protein) and about 35 mg of phosphorus. The subjects were blinded on the content of the tablets. Postprandial blood samples (~10 ml) were taken every hour, for 6 hours after meal ingestion. Blood samples were centrifuged for 15 minutes at 3500 RPM at 3°C for serum and plasma separation. Sample aliquots were stored at −80°C until analysis.

### Laboratory procedures

Concentrations of glucose, triglycerides, and total phosphate were measured using an enzymatic colorimetric method on Vitros DT 60 II Chemistry System (Ortho-Clinical Diagnostics; Johnson & Johnson, New York, NY). Serum insulin levels were analyzed using ELISA kits (Diametra Millipore, USA). Non-esterified free fatty acids concentrations were determined by enzymatic colorimetric method (Wako Chemicals, GmbH, Neuss, Germany). ELISA kits were used to analyze plasma ApoB100 (Immuno-Biological Laboratories Co., Ltd, Japan) and serum ApoB48 concentrations (Shibayagi Co., Ltd., Japan).

### Statistical analysis

Data in Table 1 represent the values derived from the first experimental session and these were almost identical to those in the second session. Changes in different parameters were analyzed using 2-factor repeated-measures ANOVA. The 2 explanatory variables being first the type of treatment (P or placebo) which was given repeatedly to the same subjects, and second the time of assessment of the dependent variable, i.e. the change in each parameter like Apo B48, at every hour after any intervention. The level of significance alpha was fixed at 0.05.

Data in tables represent the means ± SEM. P-values < 0.05 were considered statistically significant.

## Findings

**Figure 1:**
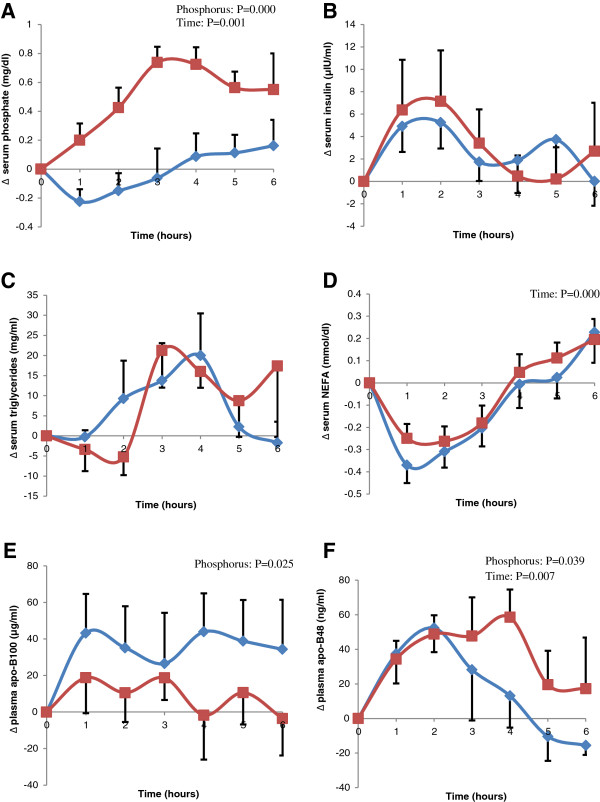
**Difference from baseline (Δ) of postprandial: serum phosphate (A), insulin (B), triglycerides (C), NEFA (D), apoB100 (E), and plasma apo-B48 (F) after the ingestion of the high fat meal with placebo (diamond) or with 500 mg P (square).** Two-factor repeated-measures analysis of variance (Phosphorus, Time and their Interaction, blue diamond; red square).

All subjects had normal BMI, waist circumference, as well as normal levels of fasting serum glucose, insulin, HOMA, TG, Total cholesterol, LDL and HDL levels (Table 1). Postprandial changes (Figure 1) in serum P were significantly different according to time (p < 0.000) and treatments (p < 0.001), while that of NEFA was significant according to time (p < 0.000) only. The pattern of changes in serum insulin and TG level varied slightly but not significantly between the two treatments.

Ingestion of P with meal resulted in a significant reduction in the postprandial changes of serum ApoB100 (p < 0.05) compared to control. Postprandial changes in ApoB48 were significantly different according to time (p < 0.01). The addition of P to meal was able to prolonged the increase in postprandial ApoB48 levels and this resulted in a statistical significant difference between the two treatments (p < 0.05).

## Discussion

Our study showed that phosphorus addition to a high fat meal increases the postprandial level of ApoB48 and concomitantly decrease that of ApoB100 of healthy male subjects. Phosphorus is known to be involved in carbohydrate, fat, protein and energy metabolism and is a component of several compounds including phospholipids. The fact that P improves insulin sensitivity implicates it in PPL since the production, clearance and degradation of TRLs are known to be affected by insulin [4,15]. Insulin resistance and diabetes stimulate the production of both intestinal (chylomicrons) and hepatic (VLDL) derived lipoproteins, and decrease their catabolic and clearance rates [4]. However, the similarity in insulin, TG and NEFA between treatment groups argues against their involvement in the observed ApoB100 and ApoB48 changes.

The similarity between treatments in serum ApoB48 during the first two hours implies that P ingestion did not affect fat absorption but rather extended the release of ApoB48 into circulation. In the P ingested group, the pattern of postprandial changes in serum P resembles that of ApoB48 (r = 0.407, p = 0.002) and this may raise the possibility of the involvement of increased intestinal P availability with ApoB48 production, especially since P is known to be well absorbed along the entire intestinal tract [16]. The similarity in postprandial insulin levels between treatments does not support a difference in chylomicron (ApoB48) clearance. On the other hand, the sustained increase in ApoB48 concentrations is believed to augment its atherogenic potential, especially since postprandial arterial lipoprotein retention prefers ApoB48 as compared to ApoB100 containing lipoproteins [17]. Postprandial VLDL production is known to start 3–4 hour post meal ingestion and thus the modest reduction in ApoB100 (component of VLDL and LDL) may imply that P content of the meal altered VLDL and LDL clearance. Such alteration may partially be related to a competition with chylomicron for clearance, especially since postprandial ApoB48 of the P ingested group was maintained for longer duration. It is worth mentioning that in the USA, daily phosphorus intake (mean ± SE) was reported to be 1277 ± 12.8 mg [18] and is mainly derived from animal sources, especially dairy products and meats. A 500 mg of phosphorus can be obtained from consuming about 500 ml of milk, 150 g of cheese, 300 g of meat or 5000 ml of carbonated cola beverages. Our study has limitations, mainly the small sample size. Even though the cross over design may be more suitable than parallel group trials for a small sample size ensuring comparability of subjects to themselves and thus less variability, we think that a study with a bigger sample size would be needed to make stronger statistical and clinical implications. At the same time, a study is needed to assess the impact of P ingestion on women. Another possible limitation is the absence of blinding of the researchers, but this may not be as important as the main outcome was objective measurements of biologic parameters.

In conclusion, in contrast to our proposed hypothesis, increased P content of a high fat meal was not able to stimulate lipid clearance from circulation. However, this study shows that P ingestion was able to manipulate several components of PPL, through the reduction in ApoB100 and the increase in ApoB48. The impact of such manipulation on the development of atheroma is not clear since both are involved in this process, although ApoB48 has a higher atherogenic effect.

## Abbreviations

PPL: Postprandial lipemia; TRLs: Triglyceride-rich lipoproteins; CM: Chylomicrons; VLDL: Very low density lipoproteins; CVD: Cardiovascular disease; TG: Triglyceride; HDL: High density lipoproteins; NEFA: Non esterified fatty acids; Apo-B48: Apolipoprotein B48; Apo-B100: Apolipoprotein B100.

## Competing interests

The authors declare that they have no competing interests.

## Authors’ contributions

OO designed the study; OO and JH conducted the research experiment; JH and DS performed the biochemical analytical methods; SH; HG; MB and OO performed the statistical analysis and wrote the paper. All authors read and approved the final manuscript.
